# Micro-Drilling of Sapphire Using Electro Chemical Discharge Machining

**DOI:** 10.3390/mi11040377

**Published:** 2020-04-03

**Authors:** Chao-Ching Ho, Jia-Chang Chen

**Affiliations:** Graduate Institute of Manufacturing Technology and Department of Mechanical Engineering, National Taipei University of Technology, Taipei 10608, Taiwan; t105568401@ntut.org.tw

**Keywords:** electrochemical discharge machining, sapphire machining, coaxial-jet, electrode wear

## Abstract

Electrochemical discharge machining (ECDM) refers to a non-traditional machining method for performing effective material removal on non-conductive hard and brittle materials. To increase the ECDM machining efficiency, traditionally, the method of increasing the machining voltage or increasing the electrolyte concentration is used. These methods can also cause overcut reaming of the drilled holes and a rough surface on the heat affected area. In this study, an innovative combinational machining assisted method was proposed and a self-developed coaxial-jet nozzle was used in order to combine two assisted machining methods, tool electrode rotation and coaxial-jet, simultaneously. Accordingly, the electrolyte of the machining area was maintained at the low liquid level and the electrolyte was renewed at the same time, thereby allowing the spark discharge to be concentrated at the contact surface between the front end of the tool electrode and the machined material. In addition, prior to the machining and micro-drilling, the output of the machining energy assisted mechanism was further controlled and reduced. For the study disclosed in this paper, experiments were conducted to use different voltage parameters to machine sapphire specimens of a 640 μm thickness in KOH electrolyte at a concentration of 5 M.

## 1. Introduction

Technology industries develop rapidly, and electronic products continue to develop in the direction of compact size with high performance. In addition, to cope with the global trend of energy saving, the demand for energy-saving lighting products has also increased significantly. Sapphire has the advantages of excellent heat conductivity [1], non-electrical conductivity [2], wide light transmittance wavelength [3], high hardness [4], high melting point [5], resistance to corrosion [6], etc., and it can be used for high/low temperature laboratory observation windows [7], optical lenses requiring high surface scratch resistance (such as cameras’ front lenses, premium watch glass, and optical finger identifier glass), etc. Sapphire can have surface epitaxy with gallium nitride (GaN) capable of emitting light in order to be used as a substrate for light emitting diodes (LEDs) [8], and its cost is lower than the LED substrates of gallium arsenide (GaAs), silicon carbide (SiC), etc.; therefore, its applicability is increased significantly [9].

As sapphire has the characteristics of non-electrical conductivity and cannot be cut easily [10], the present machining methods adopted in the industry, such as abrasive machining [11], laser machining [12], ultrasonic machining [13], and chemical etching techniques [14], all have certain drawbacks and limitations. The use of laser machining and ultrasonic machining methods to perform micro-machining on glass can cause the problems of surface cracks, degradation of the surface cleanliness, etc. Traditional machine abrasive machining is limited to the cutting of simple structures [15]. The material removal rate of chemical etching of hydrogen fluoride (HF) is relatively slow and has the issue of environmental pollution [16]. Accordingly, the method of electrochemical discharge machining (ECDM) is capable of performing effective material removal machining on non-electrically conductive hard and brittle materials. This is a non-traditional machining method [17,18] that is extremely suited to performing highly efficient micro-machining on sapphire. However, regarding the ECDM method, during the machining process, for the machining electrode and machined workpiece, the electrolyte circulation, electrolyte concentration maintenance, discharge of machining wastes, etc., can become more difficult as the machining depth increases. These phenomena can cause severe hindrances [19] of micropore machining with high precision.

In 1998, Gautam et al. [20] used a gravity fed spindle, and this rotational laboratory device was able to perform ECDM on glass materials. The experiments were conducted using two types of machining methods: rotating spindle and fixed spindle. The experimental results proved that the use of a spindle was able to allow the tool electrode to rotate such that the machining efficiency of ECDM can be effectively improved. In addition, as the discharge generated by the tool electrode rotation was not concentrated at a particular area, the wear of the tool electrode was reduced.

In 2001, Yang et al. [21] studied the feasibility of using electrolytes of the solvents of KOH, NaOH, H_2_SO_4_, NaNO_3_, NaCl, and NaClO_3_ in ECDM. The results indicated that the use of H_2_SO_4_, NaNO_3_, NaCl, and NaClO_3_ were able to generate harmful gases during the electrochemical discharge reaction process and the material removal rate was poor, and thus unsuitable for ECDM. After the experimental result comparison, they found that since the electro mobility (7.62 × 10^−8^ m^2^s^−1^V^−1^) of K^+^ was higher than the electro mobility (5.19 × 10^−8^ m^2^s^−1^V^−1^) of Na^+^, and KOH solution had a lower viscosity than that of the NaOH solution, it was able to facilitate the electrolyte flow circulation at the machining area such that when the KOH solution was used as the electrolyte, it demonstrated superior results in the machining efficiency and machining quality.

In 2014, Jiang et al. [22] used finite element analysis to compare the column electrode and conical electrode current density distributions, and found that the conical electrode current distribution concentrated at the tip portion, and the machining energy concentrated at the tip. In addition, the electrolyte circulation effect was found to be better than the one of the column electrodes, and was able to improve the machining efficiency and reduce the problem of side wall discharge. In 2015, Jiang et al. [23] used the electrolyte concentration, the electrode diameter, and the electrode immersion depth into the electrolyte as the variables to perform simulation through the finite element analysis method, and discharge machining threshold currents of ECDM were obtained. The result proved that when the electrode immersion depth into the electrolyte was shallower, the machining threshold current was greater, and the machining energy was greater.

In 2015, the Master’s thesis [24] by Sen-Fu Chung at National Central University discussed the feasibility of the use of ECDM to perform machining on sapphire materials. The experimental results indicated that when a machining voltage of 80 V was used with a tungsten carbide tool electrode (with a diameter of 350 μm and a rotational speed of 300 rpm in an electrolyte mixture of phosphoric acid and sulfuric acid, where sulfuric acid accounted for 55.56% of the mixture), in order to perform machining on the sapphire, the maximum machining depth could reach 24.4663 μm. The machining efficiency was higher than the machining efficiency of the wet etching method used by the industry for machining sapphire.

The findings from the literature [19,23,25] demonstrated that the tool electrode rotation provided a higher material machining efficiency of ECDM and aided in the improvement of the hole circularity [26]. However, the tool rotation resulted in a larger surface contact between the tool tip and the workpiece and the hole size increased from the required hole diameter [27]. Thus, overcutting and cracking [28] could be observed at the exit of the hole. In our previous research [29], we showed that the assist nozzle can prevent the effect of the discharge around the sidewall of the machining tool and reduce stray electrolysis. However, the hole circularity was significantly affected by the assisted nozzle.

In ECDM, if the method of an increased machining voltage and an increased electrolyte concentration is used, it can still achieve the objective of increasing the material removal rate. It can also cause over cutting and adverse heat impacts on the machined material due to the machining energy, resulting in the reduction of the machining quality and machining precision. Accordingly, in this research, an innovative combinational machining assisted method was proposed in order to use a self-developed coaxial-jet nozzle to allow the ECDM to combine with two assisted methods of tool electrode rotation and coaxial-jet at the same time.

## 2. Methodology

**Figure 1:**
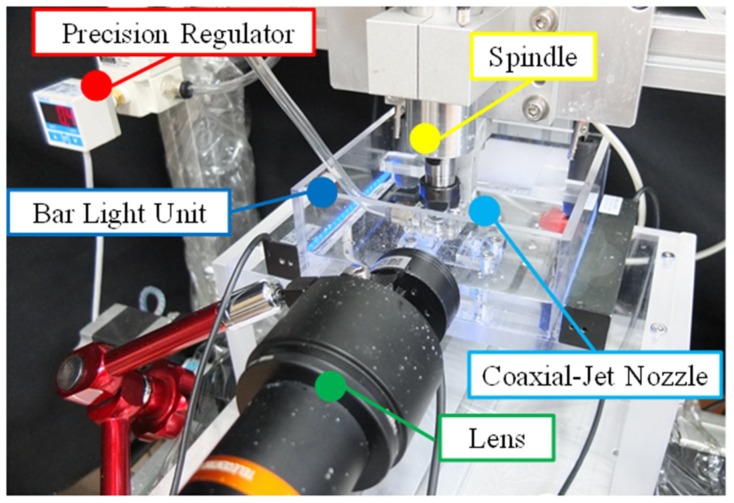
The experimental system architecture.

In this research, the experimental system architecture is as shown in Figure 1. The industrial camera can be used to observe the spark discharge condition during the experimental process and is able to directly use the hole characteristic images obtained directly from the machining in order to determine the quality of the machining for further micro-adjustment of the parameters. Consequently, there is no need to repetitively remove and clean the specimens numerous times in one experiment, followed by using an offline method to examine and determine the quality of the machining.

### 2.1. Electrode Rotation Combined with the Coaxial Jet Assisted Mechanism

**Figure 2:**
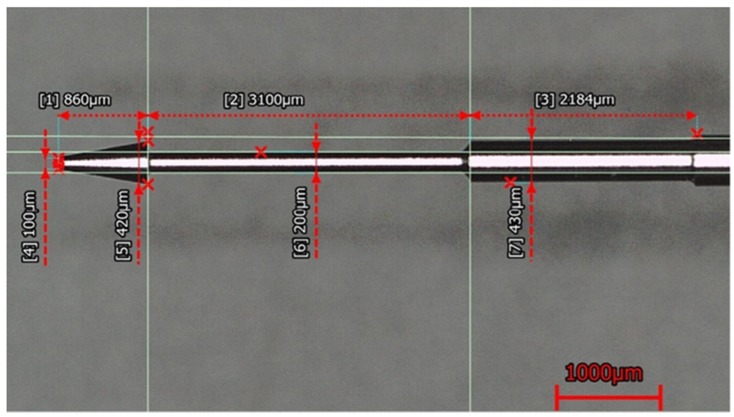
Machining electrode used in the experiments of this research.

The experiment utilized precision-pressure-regulating valves to maintain the air pressure delivered into the coaxial-jet nozzle at the pressure gauge of 0.2 kPa during the machining process, and it also used a conical electrode, as shown in Figure 2, clamped on a spindle of a rotational speed of 300 rpm such that a constant machining voltage was used to perform machining on the quartz glass specimen of a thickness of 640 μm immersed in KOH electrolyte with a concentration of 5 mol/L at an immersion depth of 1 mm.

**Figure 3:**
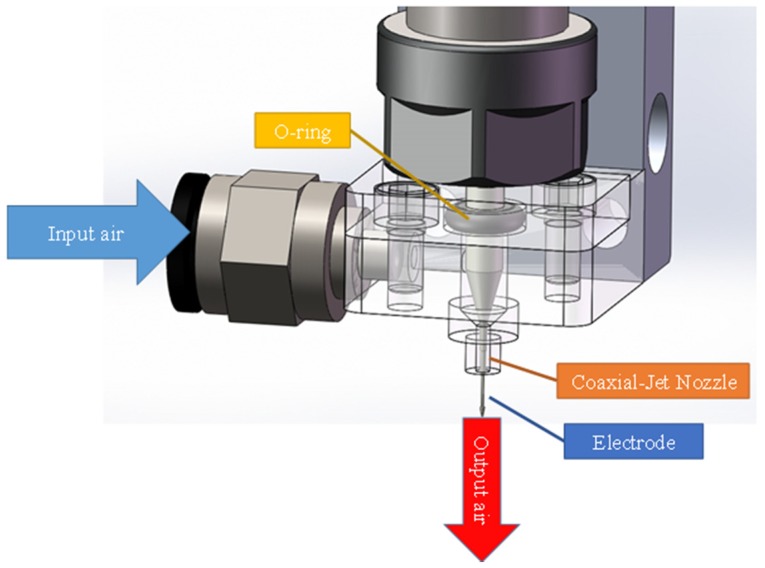
The isometric view of the self-developed and designed coaxial-jet nozzle.

The self-developed and designed coaxial-jet nozzle, as shown in Figure 3, allowed the ECDM to combine with two assisted methods of tool electrode rotation and coaxial-jet at the same time to increase the machining precision. The overall structure of the proposed nozzle was used to supply the coaxial-jet and was equipped with one gas inlet and one gas outlet for gas flow. The pressurized air gas was delivered via an inlet into the gas chamber of the main body. The ANSYS finite element analysis method was used to confirm that low pressure airflow was ejected out coaxial to the tool electrode. In the numerical analysis, the converged results were obtained for the inlet pressure of 0.2 kPa and ambient pressure of 1 atm. When the input pressure was adjusted to 0.2 kPa, the electrolyte level height was reduced to between 0.5 and 0.6 mm. In addition, when the input pressure exceeded 0.2 kPa, the electrolyte level became too low, causing poor electrolyte circulation. Consequently, in this research, 0.2 kPa was used as the input pressure for the nozzle. The experimental parameter settings in this research are as shown in Table 1.

### 2.2. Machining Electrode Wear Measurement Experiment

**Figure 4:**
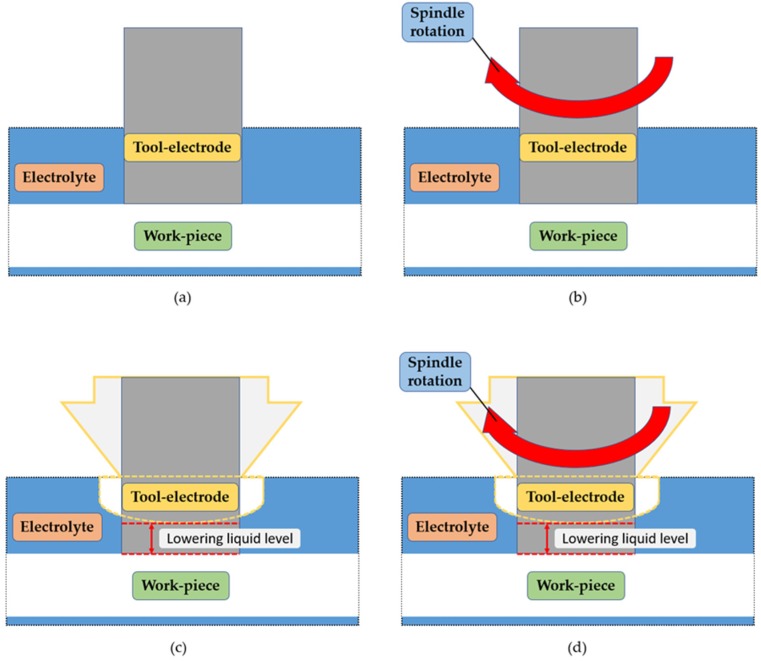
Machining method schematic views: (**a**) fixed electrode machining; (**b**) electrode rotation machining; (**c**) jet assisted machining; and (**d**) combinational assisted machining.

In this experiment, a total of four machining methods were used, including the fixed electrode machining method, electrode rotation machining method, jet assisted machining method, and electrode rotation combined with coaxial-jet assisted machining method. During the experiment, a voltage of 50 V was used for the machining of quartz glass of a thickness of 1.1 mm in KOH electrolyte at a concentration of 5 M. During the experiment, the electrode machining depth was limited to 0.9 mm in order to simulate the blind hole machining process. In addition, when the machining time reached 40 s, the system shut down the power and the electrode was lifted up. For each round of machining, a new electrode was added, thereby reducing and controlling the experimental variables. The four machining methods are shown in Figure 4.

## 3. Experimental Result

### 3.1. Machining Electrode Wear Measurement Experimental Results

**Figure 5:**
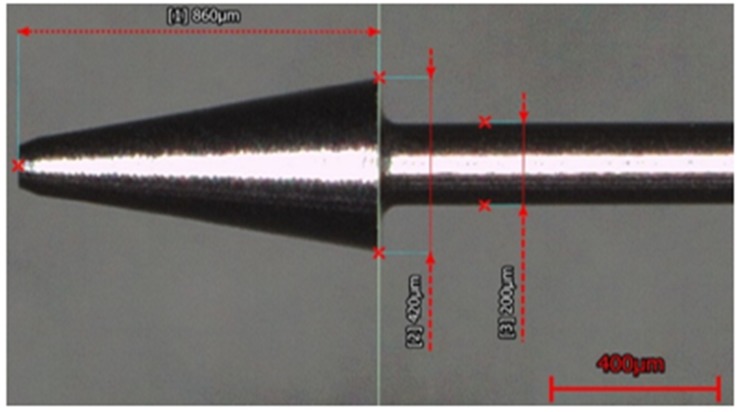
Schematic view of the original dimensions of the new electrode.

**Figure 6:**
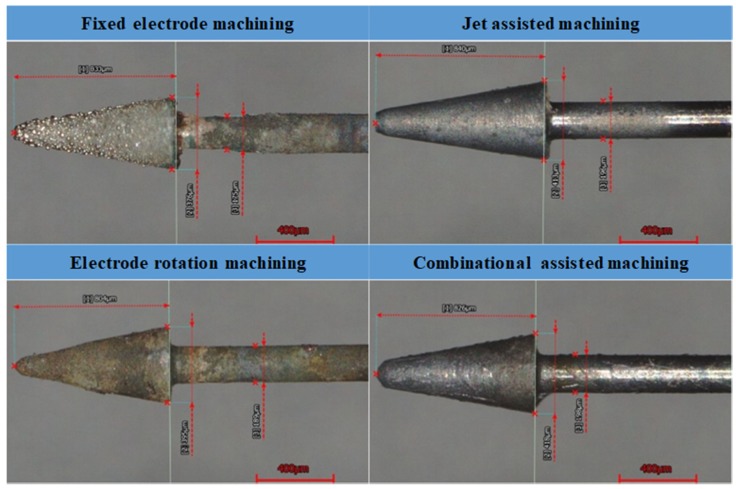
Electrode wear conditions of the different machining methods.

From the observation of the experimental data of Figure 5 and Figure 6, and Table 2, we found that the during the use of the electrode rotation machining assisted mechanism, under the effect of the mechanical rotating force, the wear of the electrode dimension in [9] was relatively greater. When the electrode rotation machining assisted mechanism was not used, since the electrolyte circulation is poor and the discharge mostly occurs as side wall discharge, the wear of dimension in [30] was greater than the wear of dimension in [15]. From the measurement results comparison with the method using the jet assisted machining mechanism, the result of the method without the use of the jet assisted machining mechanism indicated that it could effectively reduce the electrode wear. Regarding the use of the coaxial-jet assisted mechanism, regardless of whether it is used independently or used in combination with others, the result indicated that the method had a significant suppression effect on the reduction of electrode wear.

### 3.2. Sapphire Machining Result

In the experiment disclosed in this section, the method of electrode rotation combined with coaxial jet-assisted ECDM was used for the machining of sapphire in KOH electrolyte at a concentration of 5 M with the machining voltage as the variable. In this experiment, the electrode rotational speed was limited to 300 rpm, and when the machining time reached 90 s, the system executed a power shutdown, and the electrode was lifted. For each time of machining, a new electrode was replaced in order to reduce and control the experimental variables. Through the use of a three-dimensional profile measurement instrument to perform measurements on a machined sapphire specimen, machining depths of different machining voltages were obtained according to the measurement data, and the data are shown in Table 3.

### 3.3. Machining Electrode Wear Measurement Experiment

**Figure 7:**
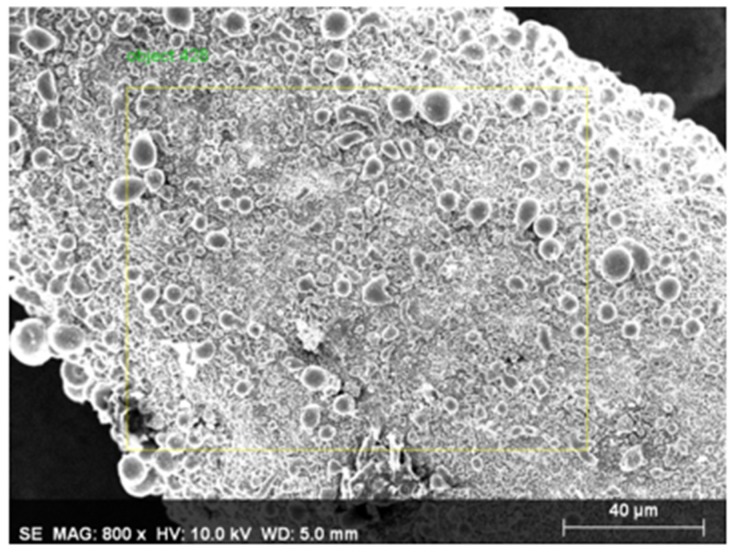
The energy dispersive X-ray spectrometer (EDS) face sampling area (used electrode).

**Figure 8:**
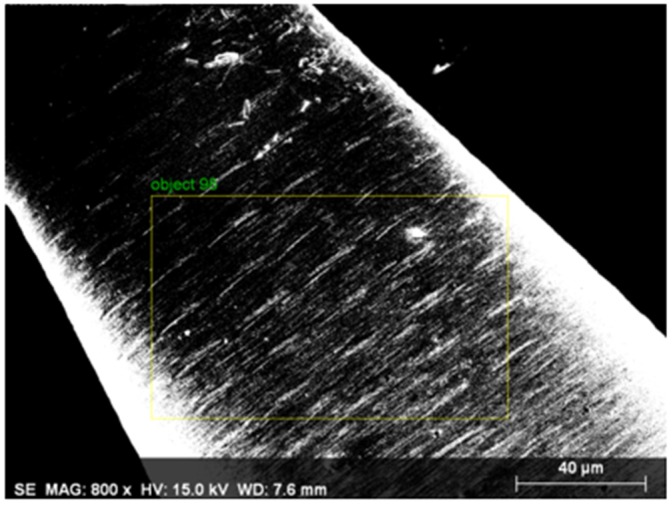
The EDS face sampling area (new electrode).

Energy dispersive X-ray spectrometer (EDS) analysis was performed to qualitatively evaluate the electrochemical reaction from the morphology of the materials and element distribution. For a tool electrode machined via the fixed electrode method with the use of the energy dispersive X-ray spectrometer (EDS), after performing the element distribution spectral analysis on a completely new tool electrode with the face sampling method, we found that the tungsten (W) content at 11.89% of an electrode after use was less than the tungsten content at 81.72% of a new electrode before use (as shown in Figure 7 and Figure 8, Table 4 and Table 5). Next, we used the multi-point sampling method on the material analyzed from the electrode surface after the use thereof in order to perform element distribution spectral analysis. We then found that the cobalt (Co) content in the material reached 78.82% and the tungsten content was only 1.84% (as shown in Table 6).

The tungsten (W) content at 11.89% of a used electrode was less than the tungsten content at 81.72% of a new electrode.

**Figure 9:**
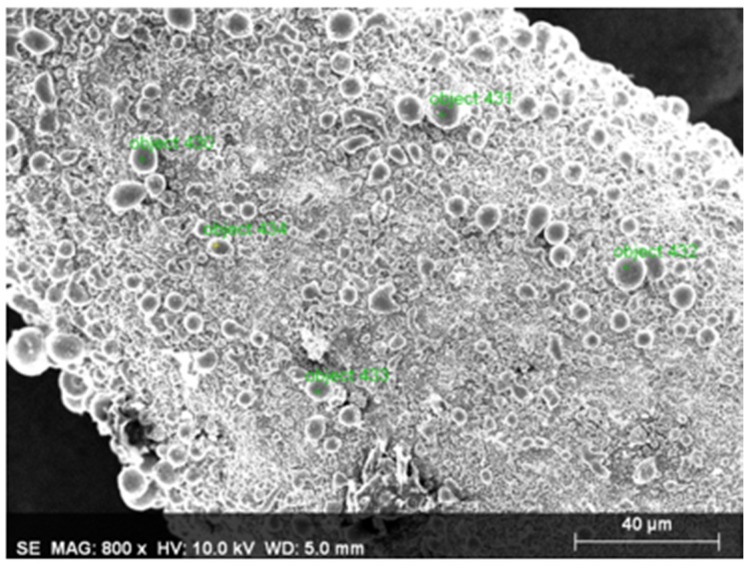
The EDS multi-point sampling area (used electrode).

As shown in Figure 9, the multi-point sampling method was used on the material analyzed from the electrode surface after use to perform element distribution spectral analysis. The cobalt (Co) content in said material reached 78.82% and the tungsten content was only 1.84% (as shown in Table 6).

The tool electrode used in this research was made from tungsten carbide powder and cobalt powder binder through the powder metallurgic method. The melting point of the cobalt element is 1768 K, the melting point of tungsten carbide is 3143 K, and the plasma temperature of the spark discharge during the machining process is higher than 5000 K [13]. Consequently, the powder metallurgic structure of the electrode becomes loose during the machining process such that the tungsten carbide is lost. The EDS inspection result and analysis was able to verify this inference. In order to reduce the wear of the electrodes during the machining process, the thermal heat flux energy should be produced mainly by the spark discharges around the tip.

**Figure 10:**
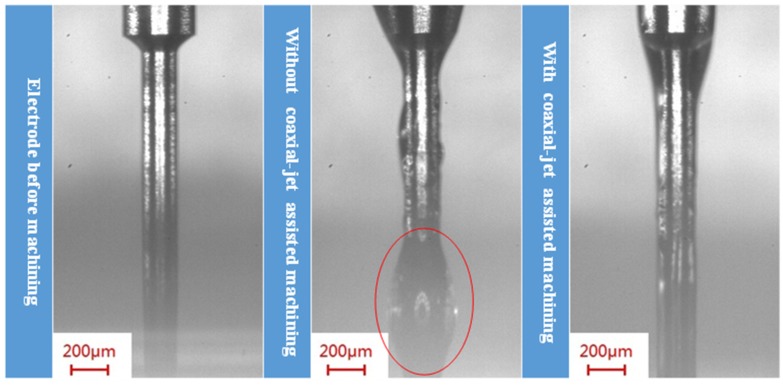
The coaxial jet-assisted method’s effect of electrolyte removal from the electrode side wall during the machining process.

Through the dynamic image (as shown in Figure 10) observation during the ECDM, we found that since the tungsten carbide electrode has excellent surface wettability, the side wall surface of the tool electrode located above the electrolyte had electrolyte droplets attached there during the ECDM process. During the machining process, as both the electrolyte and tool electrode were in a high temperature state, the strong alkalinity of the electrolyte could cause a corrosion effect on the tool electrode, and such phenomena could lead to the shortening of the useful lifetime of the electrode. According to the experimental images, we proved that the coaxial jet assisted mechanism effectively removed the electrolyte droplets attached to the side wall of the electrode above the electrolyte level to prolonge the useful lifetime of the electrode. 

### 3.4. Sapphire Machining Experiment

**Figure 11:**
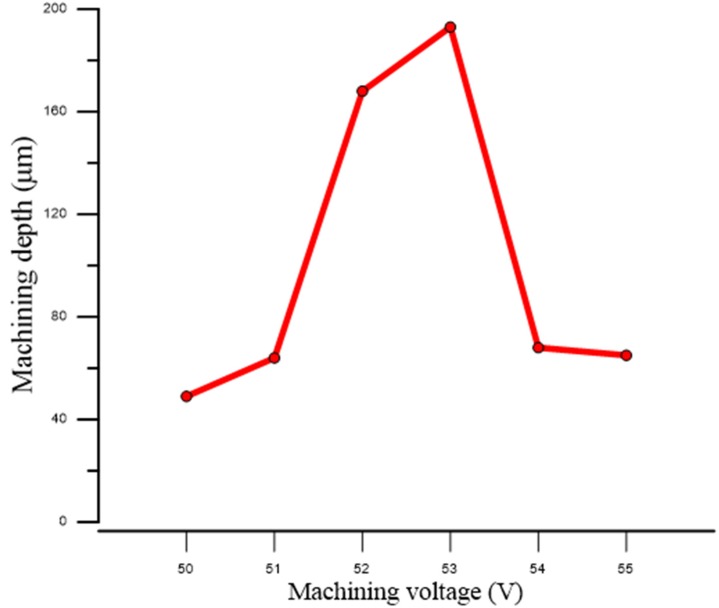
The machining voltage and machining depth relationship diagram for the use of the combinational machining method to perform machining of sapphire.

**Figure 12:**
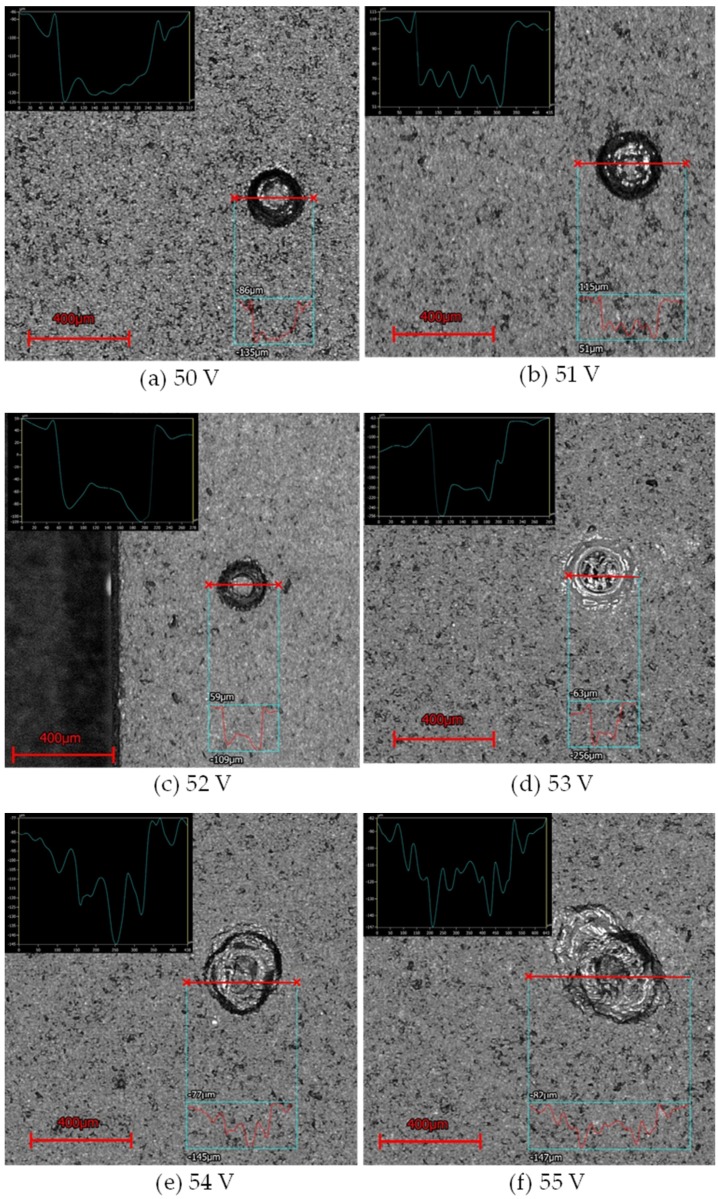
Cross sectional profile scan maps of sapphire holes machined and the hole measurement results obtained from the machining of sapphire glass with different machining voltages.

**Figure 13:**
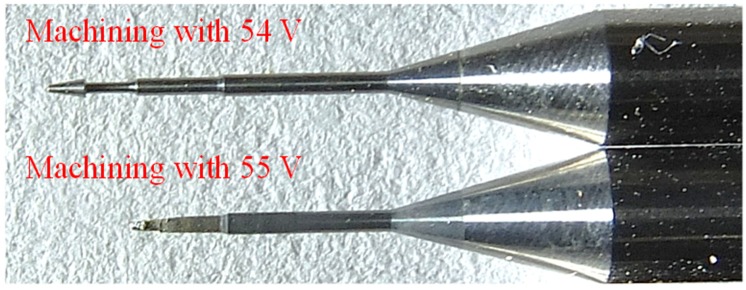
Thermal image taken during combinational assisted machining.

According to the experimental data analysis (as shown in Figure 11), we found that for the use of tungsten carbide as the machining electrode with the electrode rotation combined with coaxial-jet assisted ECDM method, the most optimal machining voltage for machining sapphire was between 52 and 53 V. As presented in Figure 12, an overly low machining voltage could cause insufficient machining energy such that the effective machining of sapphire could not be achieved. On the other hand, an overly high machining voltage could cause electrode melting, causing the machining surface to become overly rough (as shown in the machining results of 54 and 55 V in Figure 13).

## 4. Conclusions

In this paper, we investigated the machining issues related to the use of electrochemical discharge machining (ECDM) to perform non-electrical conductive hard and brittle material removal machining. To overcome the problems of overcutting, hole expansion, and rough surfaces at the thermally-affected area of hole edges that often occur in ECDM, causing degradation of the machining quality and difficulty in the improvement of precision, we proposed an innovative combinational assisted machining method to overcome the machining problems. In addition, the efficiency and precision of the assisted machining method were studied and tested in order to ensure that this method was able to overcome the machining problems of overcutting and hole expansion. We also confirmed that the method was able to improve the machining quality. 

In this study, through the machining experiments conducted on sapphire, a transparent, hard, and brittle material widely used in industrial applications, a self-developed coaxial-jet nozzle was used and ECDM was combined with two types of assisted methods of tool electrode rotation and coaxial-jet, simultaneously. Accordingly, the electrolyte of the machining area was maintained at the low liquid level and the electrolyte could be renewed at the same time, thereby allowing the spark discharge to be concentrated at the contact surface between the front end of the tool electrode and the machined material. In addition, prior to the machining and micro-drilling, an assisted mechanism for energy limitation was further adopted.

The results of this research can be summarized as follows:

1. We researched and developed an assisted nozzle capable of combining two types of assisted methods of the tool electrode rotation and coaxial-jet at the same time.
2. We established an experimental system architecture for the online observation of the ECDM experiment.
3. The combinational machining assisted method proposed in this research indicated a reduction of axial wear by 39.29% and radial wear by 84.09% in comparison to the tool electrode without assisted machining. In addition, we discussed the tungsten carbide tool electrode wear principle during ECDM.
4. The method of ECDM was used to perform the machining of sapphire with a voltage of 53 V in KOH electrolyte at a concentration of 5 M, and the machining depth reached 193 μm.

In this research, observations were made before and after the experiments, and data were collected for verification. The electrochemical discharge combinational machining assisted method proposed in this research, in comparison to the unassisted machining method, demonstrated an increased machining efficiency and a greater ability in the machining of sapphire material. Thus, the machining precision for transparent hard and brittle materials can be increased in the future, and such methods can be widely applied to the industries of material machining, optical components, semiconductors, batteries, etc., thereby achieving greater quality.

## Figures and Tables

**Table 1:** Experimental parameter settings.

Electrolyte	5 M KOH Solution
Machining voltage	50 V
Electrode immersion depth	1 mm
Machining feed pressure	0.15 kgf
Spindle rotational speed	300 rpm
Nozzle input pressure	0.2 kPa

**Table 2:** Electrode wear conditions of different machining methods.

Machining Method	(1) Dimension Wear (μm)	(2) Dimension Wear (μm)	(3) Dimension Wear (μm)
**Fixed electrode machining**	27	44	25
**Electrode rotation machining**	56	24	11
**Jet assisted machining**	20	7	4
**Combinational assisted machining**	34	2	2

**Table 3:** Machining depths with the use of combinational machining method under different machining voltages.

Machining Voltage (V)	Machining Depth (μm)	Machining Voltage (V)	Machining Depth (μm)
50	49	53	193
51	64	54	68
52	168	55	65

**Table 4:** The elemental contents of the EDS face sampling area (used electrode). El–element; AN–atomic number; Series–characteristic X-ray lines; unn. C [wt.%]–the unnormalized concentration in weight percent of the element; norm. C [wt.%]–the normalized concentration in weight percent of the element; Atom. C [at.%]–the atomic weight percent.

El	AN	Series	Net	Unn. C [wt.%]	Norm. C [wt.%]	Atom. C [at.%]	Error [wt.%]
Si	14	K-series	0	0.00	0.00	0.01	0.0
K	19	K-series	102	1.65	4.46	8.00	0.2
Cr	24	K-series	145	6.07	16.40	22.13	0.6
Co	27	L-series	1650	18.15	49.03	58.38	3.6
W	74	M-series	723	11.14	30.10	11.49	0.7
			Total:	37.02	100.00	100.00	

**Table 5:** The elemental contents of the EDS face sampling area (new electrode).

El	AN	Series	Net	Unn. C [wt.%]	Norm. C [wt.%]	Atom. C [at.%]	Error [wt.%]
Si	14	K-series	0	0.00	0.00	0.01	0.0
Cr	24	K-series	119	0.61	1.29	3.98	0.1
Co	27	K-series	345	2.56	5.42	14.74	0.2
W	74	L-series	1557	44.07	93.29	81.27	2.1
			Total:	47.24	100.00	100.00	

**Table 6:** The elemental contents of the EDS multi-point sampling area (used electrode).

El	AN	Series	Net	Unn. C [wt.%]	Norm. C [wt.%]	Atom. C [at.%]	Error [wt.%]
Si	14	K-series	0	0.00	0.00	0.01	0.0
K	19	K-series	77	0.87	2.54	3.86	0.1
Cr	24	K-series	115	4.64	13.56	15.49	0.5
Co	27	L-series	3034	26.77	78.22	78.82	4.6
W	74	M-series	155	1.95	5.68	1.84	0.2
			Total:	34.23	100.00	100.00	
